# Monkey Adrenal Chromaffin Cells Express α6β4* Nicotinic Acetylcholine Receptors

**DOI:** 10.1371/journal.pone.0094142

**Published:** 2014-04-11

**Authors:** Alicia Hernández-Vivanco, Arik J. Hone, Mick´l Scadden, Beatriz Carmona-Hidalgo, J. Michael McIntosh, Almudena Albillos

**Affiliations:** 1 Departamento de Farmacología y Terapéutica, Facultad de Medicina, Universidad Autónoma de Madrid, Madrid, Spain; 2 George E. Whalen Veterans Affairs Medical Center, Salt Lake City, Utah, United States of America; 3 Department of Biology, University of Utah, Salt Lake City, Utah, United States of America; 4 Department of Psychiatry, University of Utah, Salt Lake City, Utah, United States of America; University of Michigan, United States of America

## Abstract

Nicotinic acetylcholine receptors (nAChRs) that contain α6 and β4 subunits have been demonstrated functionally in human adrenal chromaffin cells, rat dorsal root ganglion neurons, and on noradrenergic terminals in the hippocampus of adolescent mice. In human adrenal chromaffin cells, α6β4* nAChRs (the asterisk denotes the possible presence of additional subunits) are the predominant subtype whereas in rodents, the predominant nAChR is the α3β4* subtype. Here we present molecular and pharmacological evidence that chromaffin cells from monkey (*Macaca mulatta*) also express α6β4* receptors. PCR was used to show the presence of transcripts for α6 and β4 subunits and pharmacological characterization was performed using patch-clamp electrophysiology in combination with α-conotoxins that target the α6β4* subtype. Acetylcholine-evoked currents were sensitive to inhibition by BuIA[T5A,P6O] and MII[H9A,L15A]; α-conotoxins that inhibit α6-containing nAChRs. Two additional agonists were used to probe for the expression of α7 and β2-containing nAChRs. Cells with currents evoked by acetylcholine were relatively unresponsive to the α7-selctive agonist choline but responded to the agonist 5-I-A-85380. These studies provide further insights into the properties of natively expressed α6β4* nAChRs.

## Introduction

Nicotinic acetylcholine receptors (nAChRs) are ligand-gated ion channels that are ubiquitously expressed throughout the central and peripheral nervous systems as well as in non neuronal tissues. These channels are composed of five individual subunits that assemble around a central pore and open to allow the passage of ions across the plasma membrane when activated by an agonist [1]. There are seventeen nAChR genes that code for the different nAChR subunits and include ten α, four β, and one δ, ε, and γ subunits; receptors composed of α1β1δε/γ have only been found at the neuromuscular junction. These subunits combine in various combinations to form different receptor subtypes each having unique biophysical properties including permeability to calcium and sensitivity to ligands. Many of these receptor subtypes have been implicated in human conditions. For example, receptors containing α6 subunits are involved in disorders of the central nervous system including Parkinson's disease [2], [3], nicotine reward and withdrawal [4] and self-administration [5], [6].

There are two main α6-containing subtypes that generally contain β4 or β2 subunits and form the α6β4* and α6β2* subtypes, respectively. The α6β2* subtype is thought to be primarily expressed in the central nervous system while α6β4* receptors are primarily found in cells of the peripheral nervous system including rat dorsal root ganglion (DRG) neurons and human adrenal chromaffin cells but have also been found in mouse hippocampus [7], [8], [9]. Neither of these subtypes is widely expressed and consequently less is known about their biophysical properties and functional regulation than for other nAChR subtypes. Investigational studies of α6-containing receptors have been limited in part because they have proved difficult to express in heterologous systems [10], [11]. Nevertheless, functional expression in oocytes and some mammalian cell lines has been accomplished through the construction of subunit chimeras and concatamers [12], [13], [14], [15].

In the central nervous system, mRNA for the α6 subunit has been found in catecholaminergic nuclei of the rat brain [16], particularly in midbrain dopaminergic neurons [17], [18], and in the chick retina [19]. Functional studies have shown that the release of dopamine from dopaminergic terminals of rat [20], [21] and mouse striatal neurons [22], [23] is sensitive to α-Ctx MII, an α6- and α3-containing nAChR inhibitor. Subsequent studies using α6-selective α-Ctxs identified the α6β2* subtype as the nAChR responsible for modulating striatal dopamine release [24]. Additionally, in the ventral tegmental area α6-containing nAChRs have been shown to modulate the activity of dopaminergic neurons in the presence of ethanol or nicotine [25], [26] and α6β2* receptors have been shown to modulate the release of GABA from rat presynaptic GABAergic boutons [27]. In the peripheral nervous system, α6-containing nAChRs have been shown by electrophysiological and pharmacological analysis of recordings from rat DRG neurons [9] and human adrenal chromaffin cells [7] to contain the β4 subunit. In the present study we pharmacologically evaluated acetylcholine-evoked currents from monkey adrenal chromaffin cells and demonstrate that these catacholaminergic cells also express α6β4* nAChRs. This is the first report, to our knowledge, of electrophysiological recordings of nAChR-mediated currents from primary adrenal chromaffin cells isolated from *M. mulatta.*

## Materials and Methods

### Reagents and cRNA constructs

Stock solutions of all agonists and antagonist were made in water. ACh, choline, amphotericin-B, and N-(2-hydroxyethyl)piperazine-N’-(2-ethanesulfonic acid) (HEPES) were purchased from Sigma Aldrich (Madrid, Spain) and 5-I-A-85380 was purchased from Tocris Bioscience (Bristol, UK). The α-Ctxs were synthesized as previously described [28]. The human α3, α4, β2, β3, and β4 subunit clones were provided by J. Garrett (Cognetix Inc., Salt Lake City, UT, USA) and the α6/α3 chimera was provided by J. Lindstrom (University of Pennsylvania, Philadelphia, PA, USA) and subsequently subcloned into the pSGEM expression vector by Dr. Layla Azam (University of Utah, Salt Lake City, UT, USA). Construction of the α6/α3 subunit chimera has been previously described and consists of an α3 subunit where the first 207 amino acids of the ligand-binding domain were replaced with the corresponding α6 amino acids [10]. This chimera was used because injection of non-chimeric α6 with β2 results in few, if any, functional receptors [10], [13], [29]. However, injection of β2 and β3 cRNA in conjunction with the α6/α3 chimera produces sufficient numbers of receptors for electrophysiological recordings.

### Ethics statement

Experimental procedures for obtaining monkey and mice adrenal glands were approved by the Committee for Research Ethics of the Universidad Autónoma de Madrid (registry # ES-280790000097) and conducted under the supervision of the Head of Animal Welfare and Health in accordance with Spanish and European guidelines (Boletín Oficial del Estado of March 18, 1988 and the 86/609/EEC European Council Directive). Monkey adrenal gland mRNA was purchased from Zyagen (San Diego, CA, USA). Oocytes were obtained from *Xenopus laevis* frogs. Procedures (protocol # 11-09008) for the care and use of *Xenopus* were approved by the University of Utah Institutional Animal Care and Use Committee.

### Generation of single-stranded cDNA

Total mRNA (DNAse treated) isolated from *M. mulatta* adrenal gland was used to generate cDNA using the High Capacity cDNA Reverse Transcription Kit (cat. # 4368814, Ambion, Austin, TX, USA) following the manufacturer's instructions. Briefly, a reaction volume of 25 μl was used and contained 1.25 μg of mRNA. The cycling conditions for the reverse-transcription were as follows: step 1, 25°C for 10 min; step 2, 37°C for 120 min; and step 3, 85°C for 5 min and were achieved using a PTC-200 peltier thermal cycler (MJ Research, Waltham, MA, USA).

### Analysis of nAChRs subunits using subunit-specific primers

Reverse-transcription polymerase chain reaction (PCR) analysis was performed using the Qiagen Taq PCR Master Mix Kit (Qiagen, Germantown, MD, USA) following the manufacturer's instructions. Briefly, 2.5 μl of cDNA template were added to the Master Mix solution containing sense and antisense primers (final concentration of 500 nM for each primer) for a total reaction volume of 50 μl. Negative controls for each reaction were performed by omission of the cDNA template and genomic DNA contamination was assessed by performing the reaction in the absence of the transcriptase. Primer sets for each nAChR subunit have been previously described and were designed to target *Homo sap*ien sequences [30], [31], [32], [33]. Each primer pair was aligned with their respective nicotinic subunit target sequence to verify compatibility with *M. mulatta* sequences. Full length sequences for *M. mulatta* CHRNA3 and CHRNB4 were not available in NCBI and thus, a sequence comparison for these mRNAs was performed using the sequences of *Papio anubis*. The antisense primers for CHRNA10 and CHRNB3 were found to be mismatched and were corrected to the corresponding nucleotide found in the *M. mulatta* sequence. All primers were synthesized by the University of Utah Sequencing and Genomics Core Facility at the University of Utah (Salt Lake City, UT, USA). The PCR was performed in a PTC-200 thermal cycler using an initial 5 min denaturation step at 95°C followed by 35 cycles of denaturation at 94°C for 30 s, 55–60°C for 30 s for annealing, 72°C for 45 s for extension, with a final extension step at 72°C for 10 min. Following PCR, the reactions were analyzed by gel (1.5% agarose wt/vol) electrophoresis, stained and visualized with ethidum bromide.

### Verification of the amplification products

To verify amplification of the correct sequence of interest, the bands were cut out from the gel, solubilized, and the products purified using a QIAquick PCR Purification Kit (cat. # 21804, Qiagen, Germantown, MD, USA) following the manufacture's instructions. Sequencing of the products was performed by the University of Utah Sequencing and Genomics Core Facility. The sequences were then subjected to BLAST analysis using NCBIs nucleotide BLAST program.

### Two-electrode voltage-clamp electrophysiology of *Xenopus laevis* oocytes

Detailed methods for conducting electrophysiological experiments of nAChRs heterologously expressed in *Xenopus* oocytes have been previously described [34]. Briefly, stage IV-V oocytes were injected at a 1∶1 ratio with cRNA encoding cloned human nAChR subunits α3, α4, α6/α3, β2, β3, and β4 and used 1–5 days after injection. The oocyte membranes were clamped at a holding potential of −70 mV and continuously gravity perfused with standard ND96 solution buffered with HEPES and stimulated with 1-sec pulses of 300 μM ACh or 100 μM 5-I-A-85380 once every min. The solution changes were controlled through a series of 3-way solenoid valves interfaced with a personal computer via a CoolDrive valve driver (Neptune Research & Development, West Caldwell, NJ, USA) and LabVIEW software (National Instruments, Austin, TX, USA). The agonist-gated currents were acquired using an Oocyte OC-725 series voltage-clamp amplifier (Warner Instruments, Hamden, CT, USA), filtered through a 5 Hz low-pass Bessel filter (model F1B1; Frequency Devices, Ottawa, IL, USA), and sampled at 50 Hz using a National Instruments USB-6009 digital to analog converter. The toxins were suspended in ND96 and either perfusion applied (for concentrations ≤1 μM) or applied in a static bath for 5 min (for concentrations ≥10 μM).

### Cell culture

Monkey chromaffin cells were obtained from two adrenal glands of a nine years old male *M. mulatta*. The isolation and culture of monkey adrenal chromaffin cells was performed as previously described for human chromaffin cells [7]. Chromaffin cells were isolated from adrenal glands obtained from 1–2 month old male C57BLK6/J mice according to previously established procedures [35]. Electrophysiology experiments were started 48 h after plating, to allow recovery of the nicotinic receptor expression after collagenase treatment [36], and were continued for 5 days *ex vivo*. The inherent difficulties of obtaining *M. mulatta* glands limited the number of experiments that could be performed.

### Electrophysiological recordings from adrenal chromaffin cells

Electrophysiology recordings were performed in the perforated-patch configuration using the patch-clamp technique. The external solution used to record nicotinic currents was (in mM): 2 CaCl_2_, 145 NaCl, 5.5 KCl, 1 MgCl_2,_ 10 HEPES, and 10 D-glucose; the pH was adjusted to 7.4 with NaOH. The intracellular solution composition was (in mM): 145 K-glutamate, 8 NaCl, 1 MgCl_2_, 10 HEPES, and 0.5 mg/ml amphotericin B; the pH was adjusted to 7.2 with KOH. An amphotericin B stock solution was prepared daily at a concentration of 50 mg/ml in dimethylsulphoxide and kept protected from light. The final concentration of amphotericin B was prepared by sonicating 10 μl of stock amphotericin B in 1 ml of internal solution. Pipettes were dipped in amphotericin-free intracellular solution for several seconds and then back-filled with freshly mixed intracellular solution containing amphotericin. Patch pipettes were pulled from borosilicate glass capillary tubes, partially coated with wax then heat polished and had resistances of 2–3 MΩ when filled with the internal solution. After seal formation and perforation, only recordings in which the access resistance of the pipette and the leak current were ≤20 MΩ and ≤20 pA, respectively, were accepted for analysis; series resistance was compensated up to 80%. A HEKA EPC10 amplifier (HEKA Elektronic, Lambrecht, Germany) was used to record agonist-evoked currents. The recordings were acquired at holding potential of −80 mV and filtered through a 4-pole Bessel filter at 2.9 kHz. The sampling frequency used was 10 kHz.

The perfusion system for drug application consisted of a multi-barreled polyethylene pipette positioned close to the cell under study. The agonist used were ACh (300 μM), choline (10 mM), and 5-I-A-85380 (100 μM) and always delivered from separate tubes. The cells were stimulated with 200 ms pulses of agonists once every three minutes. The antagonists were continuously perfused between pulses and this flow was only interrupted during agonist perfusion.

### Data analysis

Data analysis of electrophysiological recordings from monkey and mouse chromaffin cells was performed using using IGOR Pro software (Wavemetrics, Lake Oswego, OR, USA) and GraphPad Prism software (La Jolla, CA, USA). Concentration-response analyses of ACh-gated currents from *X. laevis* oocytes and all other statistical analyses were performed using GraphPad Prism. Concentration-response curves for inhibition of ACh-gated currents were generated by fitting the data to the Hill equation: % response  = 100/(1 +([toxin]/IC_50_)^nH^). The confidence intervals for the α-Ctx IC_50_ values are given in parenthesis. All statistical analyses of current amplitudes and densities are shown as the mean ± S.E.M. unless otherwise specified. Each data set was assessed for Gaussian distribution and only those that passed normality tests (α = 0.05) were analyzed for significance by comparing the percent block by the toxins to a theoretical value of 100%, i.e. control values, using a one sample t-test. Data sets that did not pass tests for normality were subjected to a non parametric alternative where indicated.

## Results

### The nAChR agonists ACh and 5-I-A-85380 but not choline, evoke currents in monkey chromaffin cells

**Figure 1:**
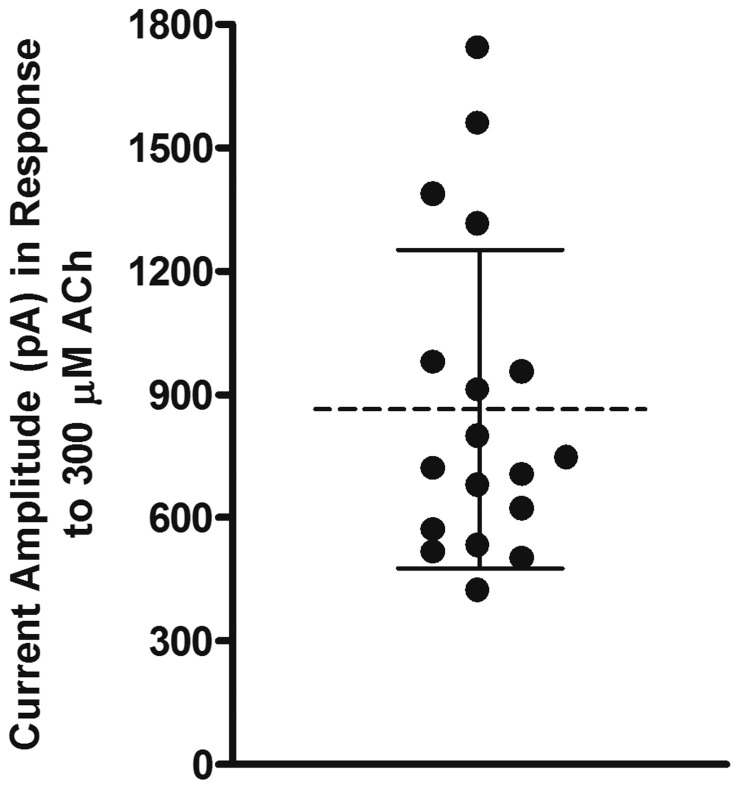
Acetylcholine evokes currents in monkey adrenal chromaffin cells. Scatter plot showing the current amplitudes evoked by 300 μM ACh. The dashed line denotes the mean and the error bars denote the S.D.M; n = 18.

**Figure 2:**
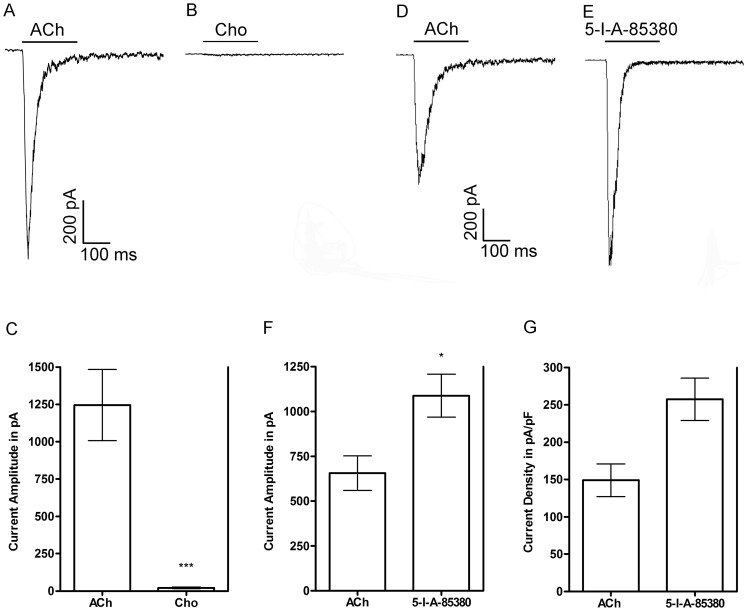
Representative trace recordings of agonist-evoked currents from monkey adrenal chromaffin cells. A and B, Current traces of ACh- and choline-evoked currents from a single cell. C, Quantitative comparison of the current amplitudes evoked by each respective agonist. Responses to 10 mM choline were compared to the average responses evoked by 300 μM ACh in the same cells; the error bars denote the S.E.M. (n = 4) and the asterisks denote statistical significance (***p<0.0001) as determined by a one sample t-test. D and E, Current traces of ACh- and 5-I-A-85380-evoked currents from a single cell. F, Quantitative comparison of the current amplitudes evoked by each respective agonist. Responses to 100 μM 5-I-A-85380 were compared to the average responses evoked by 300 μM ACh in the same cells; the error bars denote the S.E.M. (n = 4) and asterisk denote statistical significance (*p<0.05) as determined by a one sample t-test. G, The current amplitudes shown in F were normalized to membrane capacitance and shown as an expression of current density in pA/pF. The differences between the current densities produced by the two agonists were not statistically significant as determined by a Wilcoxon signed rank test (*p* = 0.0625).

We began our investigation of isolated cells obtained from the adrenal glands of *M. mulatta* monkey by testing three different nAChR agonists, ACh, choline, and 5-I-A-85380. Choline is a full agonist of heterologously expressed *M. mulatta* α7 nAChRs [37] and is considered to be α7-selective and can be used to assay for the presence of α7 receptors when multiple nAChR subtypes are potentially expressed in a given cell. The compound 5-I-A-85380 has high affinity for heteromeric receptor subtypes that contain the β2 subunit [38] and was chosen with the expectation that receptors that contain this subunit would be preferentially activated. Acetylcholine (300 μM) evoked currents with average peak amplitudes of 865 ± 387 pA (n = 18;± =  S.D.M; Fig. 1). In four of these cells, 10 mM choline evoked currents with amplitudes that were, on average, only 1.7±0.1% of the responses evoked by 300 μM ACh or 21±4 pA *versus* 1245±238 pA, respectively, in the same cells (Fig. 2A-C). The insensitivity to choline indicated that the ACh-evoked responses were mediated by a heteromeric nAChR subtype and that there were few nAChRs of the α7 subtype. Next we determined if the cells were responsive to the agonist 5-I-A-85380. As shown in Fig. 2D-F, 100 μM 5-I-A-85380 evoked currents with amplitudes that were, on average, 170±16% larger than the currents evoked by ACh or 1088±120 pA *versus* 657± 97 pA, respectively (n = 4). The current densities were also larger for those evoked by 5-I-A-85380 compared to those evoked by ACh or 256±28 pA/pF *versus* 149±22 pA/pF, respectively (Fig. 2G; n = 4). This difference was not statistically significant indicating that the effect was not due to differences in cell size. These results suggest that receptors containing the β2 subunit may also be present but at high concentrations of 5-I-A-85380 receptors containing the β4 subunit could potentially be activated [39]. The activation by both ACh and 5-I-A-85380, but not choline, suggested that the dominant nAChR subtype(s) expressed in monkey adrenal chromaffin cells contained either β2 and/or β4 subunits. However, based on the pharmacological profile of the receptors found in human and rodents, we hypothesized that these receptors were likely to be α3β4* or α6β4* nAChRs.

### PCR analysis of monkey adrenal gland mRNA demonstrates the presence of multiple nAChR subunit transcripts

**Figure 3:**
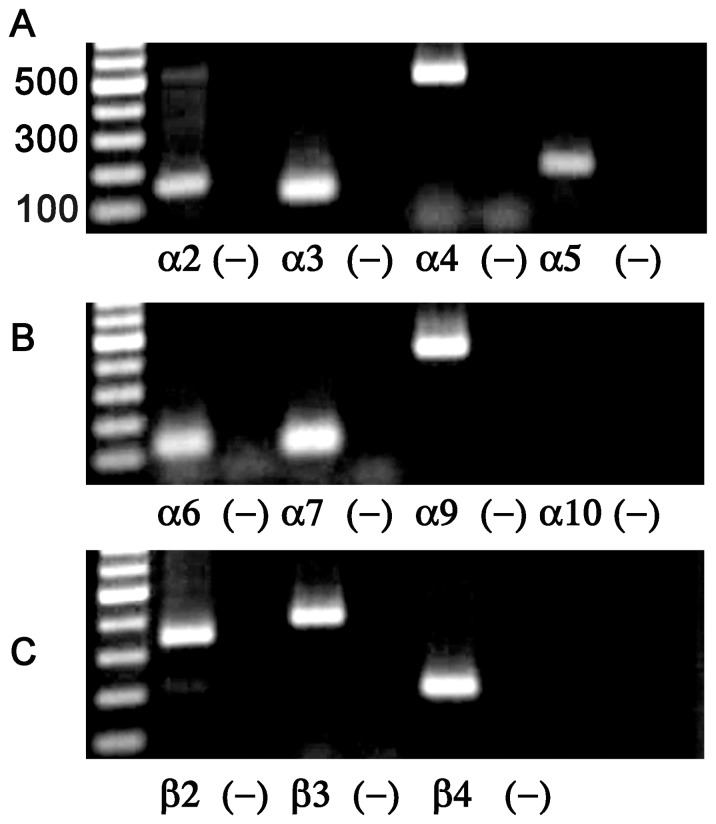
PCR analysis of the nAChR subunit transcripts present in monkey adrenal gland. A-C, PCR products for the nAChR subunits α2–α7, α9, α10, and β2–β4 were analyzed by agarose gel electrophoresis and visualized using ethidum bromide fluorescence. Negative controls for each reaction were performed by omission of the cDNA template and the results are shown in the lanes immediately to the right for each respective subunit.

nAChRs vary significantly in terms of the number of possible subtypes that a given cell may express. In rodent chromaffin cells, the predominant nAChR is the α3β4* subtype [7], [40] but in contrast, in human the predominant subtype is an α6β4* nAChR [7]. Thus, in order to guide the pharmacological analysis of the nAChR currents present in monkey chromaffin cells, we performed PCR analysis of total mRNA obtained from *M. mulatta* adrenal gland. The primers used to assay for the various nAChR subunit transcripts are provided in Table 1. As shown in Fig. 3A-C, transcripts for α6, α3, β2 and β4 were detected as well as transcripts for other nAChR subunits. Reactions performed in the absence of cDNA template were always negative (Fig. 3A-C).

**Table 1:** PCR primers.

Target	Primer Sequence	Size	Temp.	References
α2	5′-GTGGAGGAGGAGGACAGA-3′ (s)	155 bp	58 °C	[31]
	5′-CTTCTGCATGTGGGGTGATA-3′ (as)			
α3	5′-CAAGCAACGAGGGCAACG-3′ (s)	121 bp	60 °C	[32]
	5′-CCGTCCTGGCAGGGGTAG-3′ (as)			
α4	5′-GTTCCATGACGGGCGGGTGCAGTGGACT-3′ (s)	482 bp	60 °C	[32]
	5′-GGGATGACCAGTGAGGTGGACGGGATGAT-3′ (as)			
α5	5′-TTTCTTCACACGCTTCCCAAA-3′ (s)	179 bp	60 °C	[32]
	5′-TCACGGACATCATTTTCCTTCA-3′ (as)			
α6	5′-TCCATCGTGGTGACTGTGT-3′ (s)	125 bp	58 °C	[31]
	5′-AGGCCACCTCATCAGCAG-3′ (as)			
α7	5′-GGGAACCTGCTGTACATCGGC-3′ (s)	115 bp	60 °C	[32]
	5′-GGTGCTCATCGTGCGTGGG-3′ (as)			
α9	5′-GTCCAGGGTCTTGTTTGT-3′ (s)	403 bp	55 °C	[33]
	5′-ATCCGCTCTTGCTATGAT-3′ (as)			
α10	5′-CTGTTCCGTGACCTCTTCG-3′ (s)	388 bp	60 °C	Present Work
	5′-GAAGGCCGCCACGTCCA-3′ (as)			
β2	5′-CAGCTCATCAGTGTGCA-3′ (s)	347 bp	55 °C	[33]
	5′-GTGCGGTCGTAGGTCCA-3′ (as)			
β3	5′-TGGGGAGTACCTGCTGTTCA-3′ (s)	385 bp	58 °C	[30] Present Work
	5′-CGGCATAATTGGGAATACCA-3′ (as)			
β4	5′-AGCAAGTCATGCGTGACCAAG-3′ (s)	210 bp	60 °C	[32]
	5′-GCTGACACCTTCTAATGCCTCC-3′ (as)			

### α-Contoxins MII[H9A,L15A] and BuIA[T5A,P6O] distinguish among nAChR subtypes

**Figure 4:**
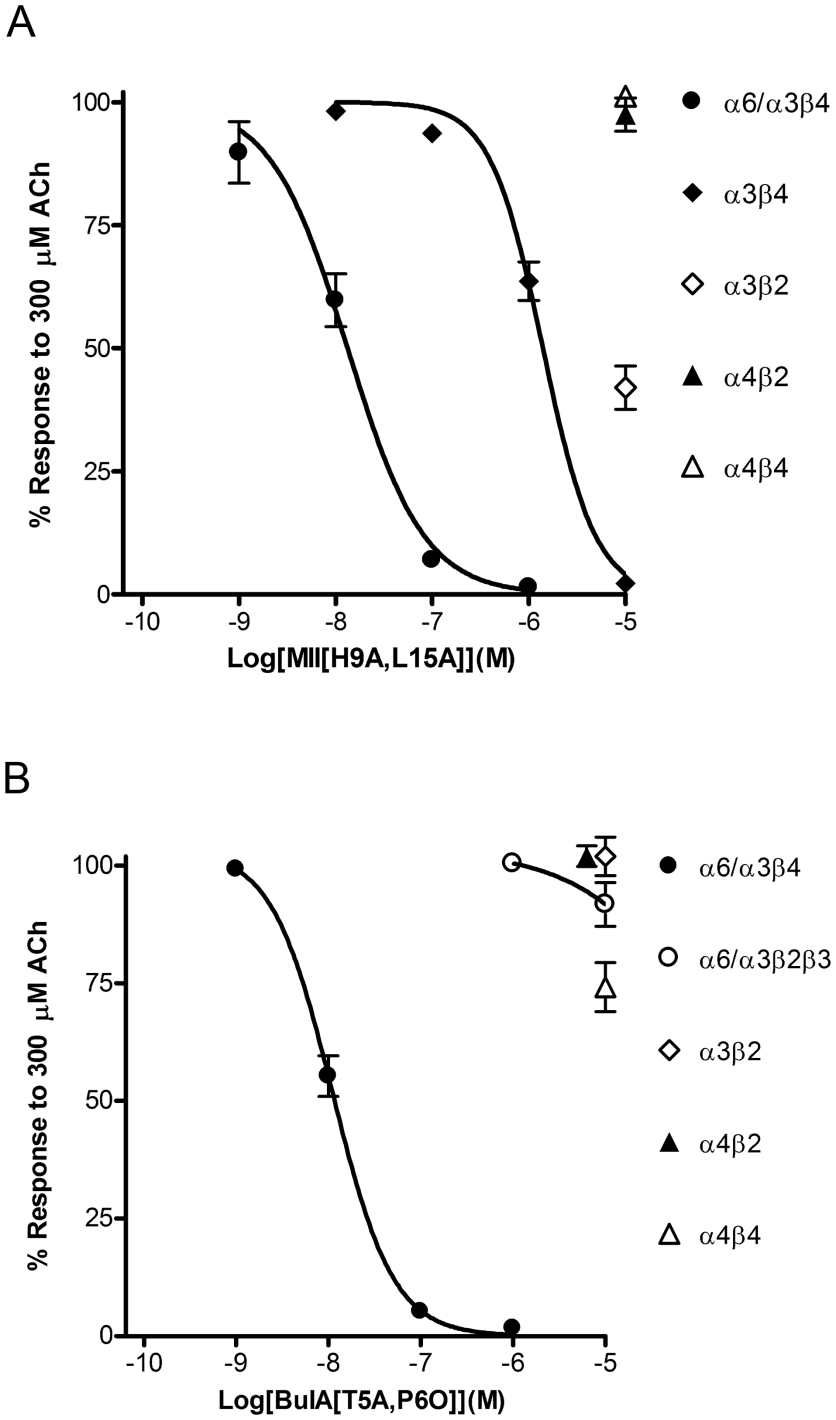
Concentration-response analysis of the inhibition of cloned human nAChRs expressed in *X. laevis* oocytes by α-Ctxs MII[H9A,L15A] and BuIA[T5A,P6O]. Oocytes expressing the indicated nAChRs were subjected to TEVC as described in “Materials and Methods” and the IC_50_ values for inhibition of the responses to ACh by each α-Ctx analog determined by fitting the data to the Hill equation. A, MII[H9A,L15A] inhibited α6/α3β4 with an IC_50_ value of 13.3 (9.7–18.1) nM (n = 4) and α3β4 with an IC_50_ value of 1.4 (1.1–1.7) μM (n = 4). Compared to controls, the responses after a 5 min exposure to 10 μM toxin were 42±4% (n = 7) for α3β2, 93±5% (n = 7) for α4β2, and 101±1% (n = 4) for α4β4 receptors. B, BuIA[T5A,P6O] inhibited α6/α3β4 with an IC_50_ value of 11.1 (9.1–13.6) nM and α6/α3β2β3 with an IC_50_ value >10 μM. The responses after a 5 min exposure to 10 μM toxin were 102±4% (n = 4) for α3β2, 102±2% (n = 4) for α4β2, and 74±5% (n = 5) for α4β4 receptors. For clarity, the symbols for inhibition of α3β2 and α4β2 receptors are shown staggered to avoid overlap.

Some α-Ctxs and their synthetic analogs show a remarkable ability to distinguish among the various nAChR subtypes. These small peptides are derived from the venom of marine cone snails that use them to capture their prey. Two of these peptides, α-Ctxs MII [28] and BuIA [41], are widely used to identify native receptors. Synthetic analogs of these α-Ctxs have been developed with increased specificity for α6-containing over α3-containing nAChRs. An analog of MII, MII[H9A,L15A], distinguishes between rat α6/α3β4 and α3β4, being more potent on the α6/α3β4 subtype [42], and BuIA[T5A,P6O], an analog of BuIA, distinguishes between rat and mouse α6/α3β4 and α6/α3β2β3 receptors, being more potent on the α6/α3β4 subtype [8]. Since the activity of these α-Ctx analogs on primate receptors had not been previously quantified, we tested them on heterologously expressed human receptors in *X. laevis* oocytes and determined their IC_50_ values. MII[H9A,L15A] inhibited ACh-evoked responses mediated by α6/α3β4 receptors with an IC_50_ value of 13.3 (9.7–18.1) nM (Fig. 4A). This value was 100-fold lower than the 1.4 (1.1–1.7) μM value obtained for α3β4 nAChRs (Fig. 4B). Next we tested BuIA[T5A,P6O] on the α6/α3β4 and α6/α3β2β3 subtypes. This BuIA analog was >900-fold more potent at inhibiting α6/α3β4 versus α6/α3β2β3 nAChRs (IC_50_ values of 11.1 (9.1–13.6) nM *versus* >10 μM, respectively). Thus, MII[H9A,L15A] can be used to distinguish between α6β4* and α3β4* nAChRs and BuIA[T5A,P6O] can be used to distinguish between α6β4* and α6β2* nAChRs. We also tested MII[H9A,L15A] and BuIA[T5A,P6O] on non α6-containing human nAChR subtypes including α3β2, α4β2, α4β4 and found that both toxins showed very limited activity on these receptors subtypes (Fig. 4A,B).

### α-Ctxs MII[H9A,L15A] and BuIA[T5A,P6O] potently inhibit ACh-evoked currents in monkey chromaffin cells

**Figure 5:**
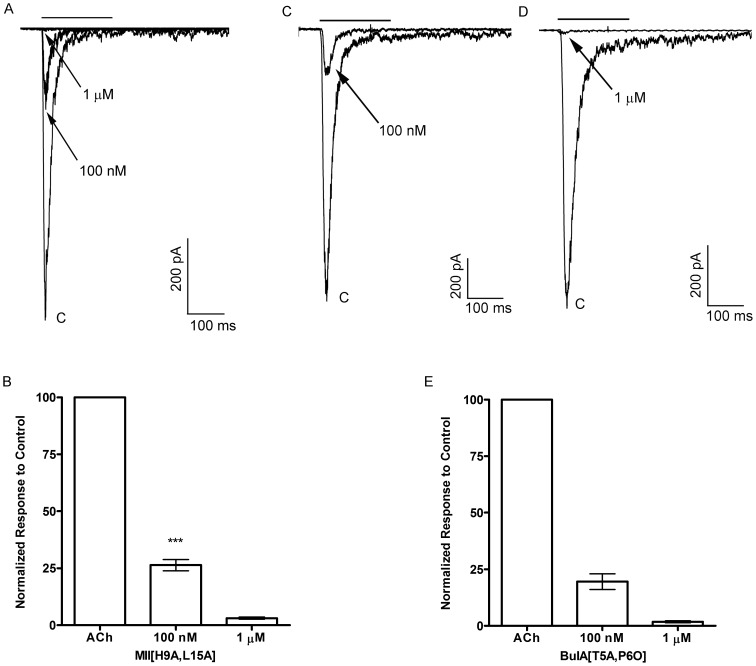
α-Ctx MII[H9A,L15A] and BuIA[T5A,P6O] potently inhibit the ACh-evoked currents in monkey chromaffin cells. A, Representative trace recordings of ACh-evoked currents and the inhibition by 100 nM and 1 μM MII[H9A,L15A]. B, Quantitative analysis of the inhibition by 100 nM (n = 5) and 1 μM MII[H9A,L15A] (n = 4). Error bars show average values ± S.E.M (n = 5); asterisks denote statistical significance (***p<0.001) as determined by a one sample t-test. C,D, Trace recordings of ACh-evoked currents and the inhibition by 100 nM and 1 μM BuIA[T5A,P6O]. E, Quantitative analysis of the inhibition by 100 nM (n = 2) and 1 μM BuIA[T5A,P6O] (n = 4); error bars show average values ± S.E.M.

**Figure 6:**
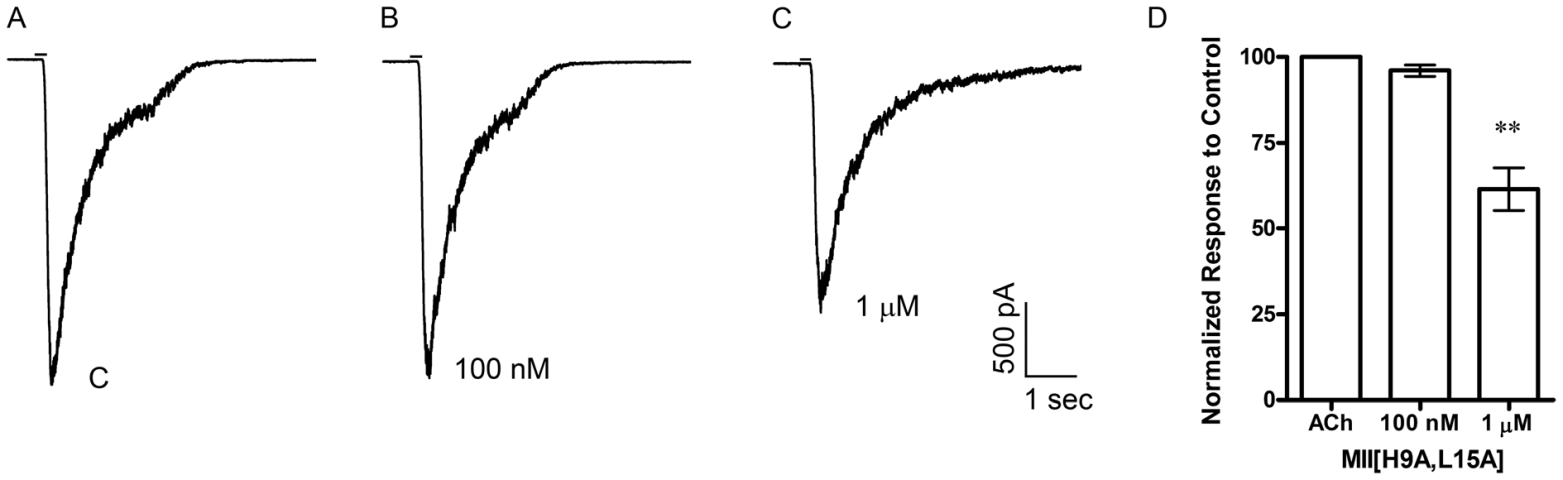
α-Ctx MII[H9A,L15A] inhibits ACh-evoked responses less potently in mouse adrenal chromaffin cells. A, Representative trace recordings of ACh-evoked currents and the inhibition by 100 nM and 1 μM MII[H9A,L15A]. B, Quantitative analysis of the inhibition by 100 nM (n = 6) and 1 μM MII[H9A,L15A] (n = 6). Statistical analysis of the inhibition by 100 nM indicated no statistical significance compared to control responses (*p*>0.05) but significance for 1 μM (*p*<**0.01); error bars show average values ± S.E.M.

To pharmacologically determine the nAChR subtype mediating the ACh-evoked currents in monkey chromaffin cells, we started by using MII[H9A,L15A] at 100 nM, a concentration where little, if any, inhibition of α3β4* receptors would be expected, followed by a concentration of 1 μM. At a concentration of 100 nM, this MII analog inhibited the ACh-evoked responses by 74±2% (n = 5) and at 1 μM the responses were almost completely abolished (97±0.4% inhibition; n = 4) (Fig. 5A,B). Next we tested BuIA[T5A,P6O], also at 100 nM and 1 μM, to selectively inhibit α6β4* *versus* α6β2* nAChRs. The ACh-evoked responses were inhibited by 81±4% (n = 2) at 100 nM and by 98±0.4% (n = 4) at 1 μM (Fig. 5 C-E). As an additional test of the specificity of MII[H9A,L15A] for the α6β4 subtype over the α3β4 subtype, we tested the toxin on mouse chromaffin cells that have been reported to lack mRNA for the α6 subunit [43] and thus the receptors expressed by these cells are likely to be mainly α3β4* nAChRs. As shown in Fig. 6, in the presence of 100 nM MII[H9A,L15A], the average response to ACh was 96±2% (n = 6) of control responses and in the same cells the responses in the presence of 1 μM were on average 62±6% (n = 6) of controls. These data indicate that the nAChRs expressed in *M. mulatta* adrenal chromaffin cells are α6β4* nAChRs.

### 5-I-A-85380 activates monkey chromaffin cell α6β4* nAChRs

**Figure 7:**
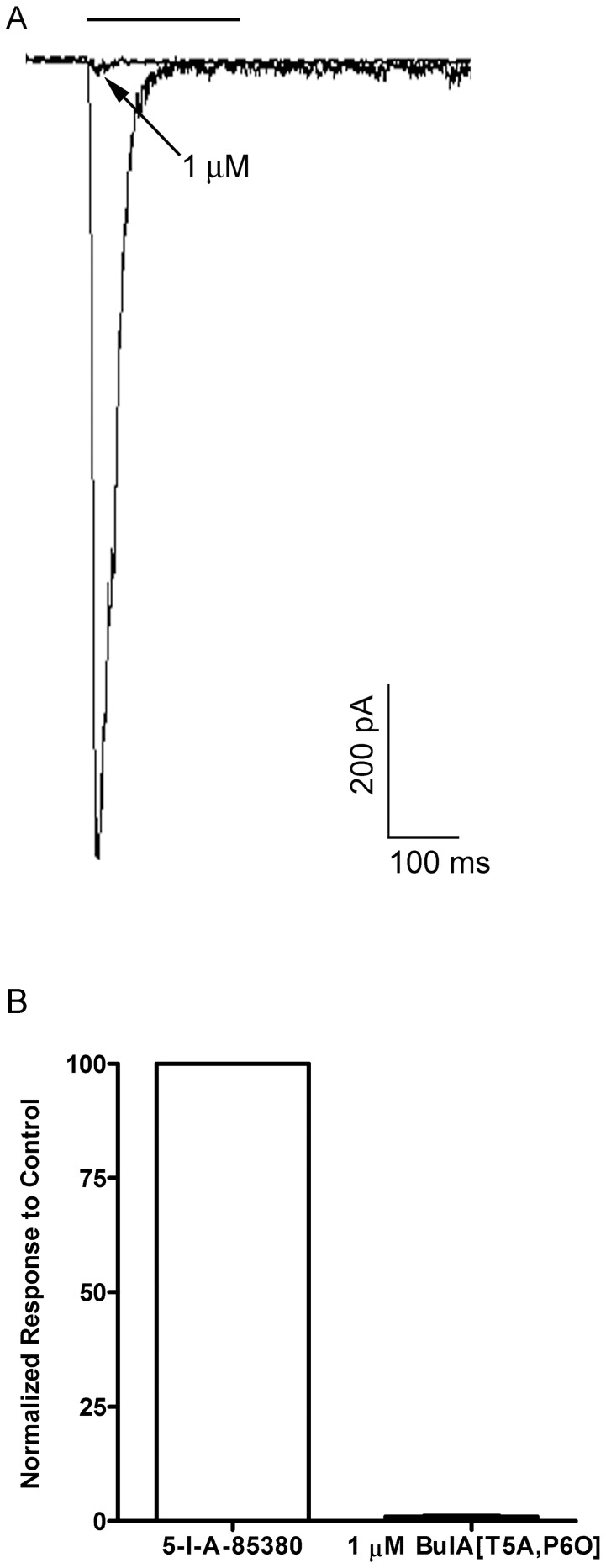
BuIA[T5A,P6O] inhibits 5-I-A-85380-evoked currents in monkey chromaffin cells. A, Representative trace recordings of 5-I-A-85380-evoked currents and the inhibition by 1 μM BuIA[T5A,P6O]. B, Quantitative analysis of the inhibition by 1 μM BuIA[T5A,P6O] (n = 4); error bars show average values ± S.E.M.

As shown in Fig. 2D-F, the efficacy of 100 μM 5-I-A-85380 in evoking currents was greater than 300 μM ACh in cells that were sequentially stimulated with both agonists. Although 5-I-A-85380 has high affinity for receptors containing the β2 subunit, at 100 μM receptors containing the β4 subunit may also be activated. To determine if 100 μM 5-I-A-85380 could activate α6β4* nAChRs, we tested this compound on heterologously expressed human α6/α3β4 receptors in *Xenopus* oocytes and found that at 100 μM this compound activated currents that were on average 100±12.1% (n = 4; Fig. S1) of the amplitude produced by 300 μM ACh in the same cells. Additionally, inhibition of 5-I-A-85380-evoked currents in monkey chromaffin cells by BuIA[T5A,P6O] would also suggest that this agonist activates α6β4* nAChRs. Indeed, perfusion with 1 μM BuIA[T5A,P6O] almost completely abolished the 5-I-A-85380-evoked currents (95±0.4% inhibition; n = 4) (Fig. 7A,B). Thus 5-I-A-85380 shows high efficacy in activating the nAChRs in monkey chromaffin cells and the inhibition of these currents by BuIA[T5A,P6O] indicated that they were mediated by the α6β4* subtype and not by nAChRs containing two α_x_β2 ligand binding sites.

## Discussion

In this work we provide the first report of the molecular and pharmacological characterization of the nAChRs expressed by monkey adrenal chromaffin cells. Initial studies demonstrated that the nAChRs in these cells could be activated by ACh and 5-I-A-85380 but not choline (Fig. 2A-F). These results suggested that the dominant receptor expressed was a heteromeric nAChR subtype. PCR analysis of monkey adrenal gland mRNA demonstrated the presence of transcripts for multiple nAChR subunits including α2–α7, α9, and β2–β4 but not α10 (Fig 3A-C). Subsequent pharmacological analysis of the agonist-evoked currents was performed using α-Ctxs that selectively target the α6/α3β4 receptor over the α6/α3β2β3 and α3β4 subtypes as determined in *Xenopus* oocytes (Fig. 4A, B). In monkey chromaffin cells, substantial inhibition of the ACh-evoked currents was achieved using a submaximal concentration (100 nM) of MII[H9A,L15A] (Fig. 5A,B), an antagonist that, as shown in Figure 4A, is >100-fold more potent on human α6/α3β4 receptors than α3β4 receptors. Substantial inhibition was also achieved using 100 nM BuIA[T5A,P6O] (Fig. 5C,E) which is >900-fold more potent on α6/α3β4 receptors than α6/α3β2β3 receptors (Fig. 4B). Complete inhibition of the ACh-evoked currents was achieved using a concentration of 1μM MII[H9A,L15A] or BuIA[T5A,P6O] (Fig. 5A,B and 5D,E). In mouse chromaffin cells, that likely only express α3β4*, much less inhibition was observed by MII[H9A,L15A] lending further support to our conclusion that monkey chromaffin cells predominantly express α6β4* nAChRs. Thus, when taken together the molecular and pharmacological evidence presented here support the presence of a nAChR in monkey chromaffin cells that contains α6 and β4 subunits and that the receptor composed of these subunits, the α6β4* subtype, predominates. We note, however, that these experiments do not rule out the possible presence of additional subunits such as α5, β2, or β3, that are known to assemble with the α6 subunit [44], or homomeric α7 nAChRs. Previously in human chromaffin cells, we found that α7 nAChRs contribute only ∼7% to the whole-cell current under electrophysiological conditions similar to those used in the current study and that positive identification was only possible using selective α7 agonists and positive allosteric modulators to prevent desensitization [45]. These compounds may also be useful in future studies to determine if α7 nAChRs are also functionally expressed in monkey chromaffin cells.

We also observed that the nAChR agonist 5-I-A-85380 at 100 μM was more efficacious than 300 μM ACh at activating monkey chromaffin cell nAChRs (Fig 2D-G) and as efficacious as 300 μM ACh when tested on human α6/α3β4 nAChRs expressed in *Xenopus* oocytes (Fig. S1). This ligand was initially described as an α_x_β2-selective ligand but was later shown to also activate other non β2-containing nAChRs [38], [39]. In this report we have shown that 5-I-A-85380 also activates heterologously expressed human α6/α3β4 as well as native monkey α6β4* nAChRs as evidenced by the fact that currents evoked by this compound in monkey cells could be fully inhibited by 1 μM BuIA[T5A,P6O] (Fig. 6A,B). The presence of multiple discrete agonist and/or antagonist binding sites in a single nAChR complex can be challenging to detect in functional assays. There are two putative agonist binding sites in a given α_x_β_x_ receptor complex and gating of the channel is thought to require the binding of two agonist molecules [1], [46]. Thus, an agonist that shows selectivity for α_x_-β2 binding sites would not be expected to activate receptors that contain both α_x_-β4 and α_x_-β2 binding sites. In contrast, a single antagonist ligand of nAChRs is sufficient for inhibition of agonist-evoked responses therefore, the simplest interpretation of the data presented here is that the nAChRs in monkey chromaffin cells contain at least one α6-β4 ligand binding interface. Nevertheless, future studies using radiolabeled ligands and immunoprecipitation to assay for the presence of additional subunits is warranted.

**Figure 8:**
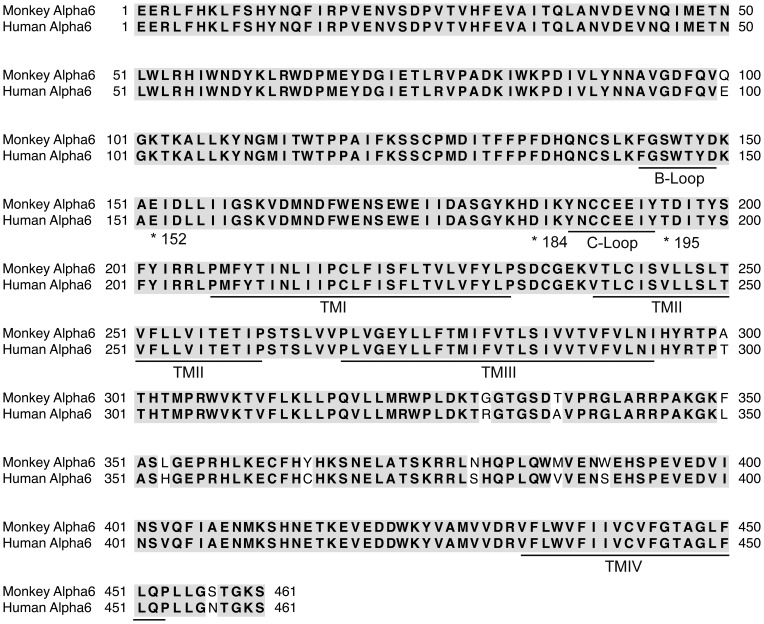
Sequence alignment of monkey and human α6 nAChR subunits. A, Sequence alignment of monkey and human α6 subunits identified a single residue in the extracellular ligand-binding domain at position 100 that differed between the two species. The asterisks identify residues that have previously shown to be important for α-Ctx binding [47], [48], [49]. Note that these residues are strictly conserved between the two species. TM indicates the transmembrane domains.

Although the α6 and α3 subunits are highly homologous and receptors composed of these subunits often have similar pharmacological properties with respect to agonist and antagonist sensitivities in some cases large differences can be observed. For example, it was recently reported that some analogs of α-Ctx PeIA show a 15,000-fold difference in the IC_50_ values for heterologously expressed rat α6/α3β2β3 *versus* α3β2 nAChRs [47]. This difference was shown to be attributed to three amino acids that differ between the α6 and α3 ligand binding domains. Differences in amino acid composition of the same subunit between species can also affect ligand potencies. A comparative study between human and monkey showed that there are minor differences in the sensitivity of heterologously expressed α7 receptors to agonists but not to antagonists even though the ligand binding domains between the two species differ by only two amino acids [37]. We performed a sequence alignment between monkey and human α6 subunits and found that in the ligand-binding domain there is a single amino acid difference at position 100 (Fig. 8); in monkey this residue is a glutamine whereas in human it is a glutamate. This single amino acid difference is not likely to affect the binding of the α-Ctxs used in this study, however, because the critical residues of the α6 subunits that interact with the α-Ctxs are strictly conserved across monkey, human, and rodent species. These residues, E152, D184, and T195, have been previously identified as the critical residues that confer high affinity binding of MII- and BuIA-related α-Ctxs to heterologously expressed α6-containing nAChRs [47], [48], [49]. Thus, the potencies obtained in this report for heterologously expressed human α6-containing nAChRs are likely to match those of native monkey α6-containing nAChRs. Indeed, the values obtained for inhibition of monkey α6β4* nAChRs by 100 nM and 1 μM MII[H9A,L15A] and BuIA[T5A,P6O] closely match the values obtained for human α6/α3β4 nAChRs in oocytes (Fig. 5A-E, and 4A,B, respectively). We also performed a sequence alignment of other human and monkey nAChR subunits and note that the sequences of the β2 extracellular ligand binding domains differ by a single amino acid at position 165 where there is a conservative aspartate to glutamate difference between monkey and human, respectively (data not shown). Unfortunately, complete sequences for *M. mulatta* α3 and β4 subunits are not available in NCBI for analysis. However, a comparison between human and baboon (*Papio anubis*) α3 revealed that the amino acids in the positions important for α-Ctx binding are also shared between these two species.

Coexpression of α6 and β4 subunits appears to be limited to a few discrete areas in the nervous system. In rodents, immunoprecipitation studies have demonstrated that α6 and β4 subunits are present in the retina [50] and studies that utilized PCR analysis showed that α6 and β4 subunits may be expressed in neurons of the ventral tegmental area and DRG neurons [9], [27], [51], [52]. In *M. mulatta, in situ* hybridization studies of brain slices found α6 and β4 coexpression in the lateral part of the medial habenula [53]. Electrophysiological recordings of α6β4* nAChR currents have only been demonstrated in rat DRG neurons [9], in human adrenal chromaffin cells [7], and in *M. mulatta* chromaffin cells as shown in the present study. In human chromaffin cells, α6β4* nAChRs are the predominant nAChR subtype expressed in contrast to what is found in rodent chromaffin cells where the nAChRs that predominate are α3β4* nAChRs [7], [40]. As in human chromaffin cells, α6β4* nAChRs also appear to dominate in monkey chromaffin cells. Thus, there is a species difference between primates and rodents in terms of nAChR expression by chromaffin cells.

Ligands that target α6β2* nAChRs have been proposed as potential pharmacological agents for the treatment of several human conditions including Parkinson's and nicotine dependence [54], [55]. However, ligands that target α6β2* would need to be devoid of activity on the α6β4* subtype to avoid potential cardiovascular side effects caused by alternations in the release of catecholamines from the adrenal gland. Our study provides valuable insights into the pharmacology of α6β4* nAChRs in catecholaminergic cells and validate chromaffin cells as a particularly promising system for the study of native α6β4* nAChRs.

## Supporting Information

Figure S1 **The activity of 5-I-A-85380 on α6/α3β4 nAChRs.** Human α6/α3β4 nAChRs were expressed in *Xenopus laevis* oocytes and subjected to two-electrode voltage-clamp electrophysiology as described in “Materials and Methods”. The responses to 100 μM 5-I-A-85380 were normalized to the responses to 300 μM ACh obtained in the same cells. The responses to 5-I-A-85380 were on average 100±12% of those evoked by 300 μM ACh. The error bars denote the S.D.M from four oocytes.
(TIF)
